# Innovative Technological Approach for the Cyclic Nutrients Adsorption by Post-Digestion Sewage Sludge-Based Ash Co-Formed with Some Nanostructural Additives under a Circular Economy Framework

**DOI:** 10.3390/ijerph191711119

**Published:** 2022-09-05

**Authors:** Piotr Sakiewicz, Krzysztof Piotrowski, Mariola Rajca, Izabella Maj, Sylwester Kalisz, Józef Ober, Janusz Karwot, Krishna R. Pagilla

**Affiliations:** 1 Division of Nanocrystalline and Functional Materials and Sustainable Pro-Ecological Technologies, Institute of Engineering Materials and Biomaterials, Faculty of Mechanical Engineering, Silesian University of Technology, Konarskiego 18A, 44-100 Gliwice, Poland; 2 Department of Chemical Engineering and Process Design, Faculty of Chemistry, Silesian University of Technology, ks. M. Strzody 7, 44-100 Gliwice, Poland; 3 Institute of Water and Wastewater Engineering, Faculty of Energy and Environmental Engineering, Silesian University of Technology, Konarskiego 18, 44-100 Gliwice, Poland; 4 Department of Power Engineering and Turbomachinery, Faculty of Energy and Environmental Engineering, Silesian University of Technology, Konarskiego 18, 44-100 Gliwice, Poland; 5 Department of Applied Social Sciences, Faculty of Organization and Management, Silesian University of Technology, Roosevelta 26-28, 41-800 Zabrze, Poland; 6 Sewage and Water Supply Ltd., Pod Lasem 62, 44-210 Rybnik, Poland; 7 Department of Civil & Environmental Engineering, University of Nevada, Reno, NV 89557, USA

**Keywords:** innovative pro-ecological technology, wastewater treatment, post-digestion sewage sludge, biogas plant, adsorption, ash, halloysite, kaolinite, nutrients, slow release, agricultural fertilizers

## Abstract

This paper presents a new, innovative technological approach, in line with Circular Economy principles, to the effective management of sludge generated during municipal wastewater treatment processes and subsequently used for biogas production. This approach allows for optimal, functional, and controlled cascade-type biotechnological thermal conversion of carbon compounds present in sewage sludge, later in solid digestate residues (after biogas production), and finally in the ash structure (after incineration, purposefully dosed nanostructural additives make the production of a useful solid product possible, especially for cyclic adsorption and slow release of nutrients (N, P, K) in the soil). The idea is generally targeted at achieving an innovative conversion cycle under a Circular Economy framework. In particular, it is based on an energy carrier (methane biogas) and direct energy production. The functionalized combustion by-products can be advantageous in agriculture. The use of ashes with nanostructural additives (halloysite, kaolinite) from combustion of sewage sludge after the anaerobic fermentation as an adsorbent of selected nutrients important in agriculture (Na^+^, K^+^, NO_3_^−^, SO_4_^2−^, PO_4_^3−^, Cl^−^) was verified at laboratory scale. The tests were carried out both for pure ash and for the ash derived from combustion with the purposeful addition of kaolinite or halloysite. The equilibrium conditions for nitrate, potassium, sodium, phosphate(V), sulphate(VI), and chloride ions from aqueous solutions with the use of the three adsorbent structures were determined. The obtained innovative results were interpreted theoretically with adsorption isotherm models (Langmuir, Freundlich, Temkin, Jovanović). The most spectacular and clearly favorable results related to the influence of nanostructural additives in the process of sludge combustion, and formation of sorption surfaces under high temperature conditions were identified in the case of sorption-based separation of phosphate(V) ions (an increase from 1.13% to 61.24% with the addition of kaolinite, and even up to 76.19% with addition of halloysite).

## 1. Introduction

Global economic development is accompanied by a dynamic increase in the role of pro-ecological technologies and related renewable energies, which are dependent, among others, on the effective management of wastewater resources [1,2,3,4]. Until now, a large fraction of the waste from anaerobic digestion (biogas production) has not been used further. Reasons were, among others, fear of too large a single dose of fertilizer, which may indirectly intensify the effects of leaching of nutrients and cause intense eutrophication. This resulted in EU regulations regarding the release of nitrogen into the environment (the nitrogen act). Another problem was the difficulty of storing the post-fermentation residues, which were subject to further uncontrolled processes outside the fermentation chamber to adapt their distribution to the dates of cyclical agricultural works. It results in spontaneous emission of methane, carbon dioxide, and odor compounds, unfavorable for the biosphere. It should also be emphasized, that in the case of municipal wastewater, a large part of the substrates of the anaerobic fermentation process is lignocellulose from fiber and toilet paper. Due to its biological structure, it is characterized by a long bioconversion time.

Modern treatment technologies enable effective purification of complex municipal wastewater mixtures. After all, some innovative technological solutions are still sought after, in particular with regard to the effective management of the resulting by-products and thus the development of possibly waste-free technological processes. For example, in this case the management of post-technological sludge according to Circular Economy principles is a key element of the design work. It is related to the construction, modernization, or reconstruction of existing multifunctional treatment installations. The detailed optimization of the complex, multi-stage integrated process of municipal wastewater treatment combined with biogas production was presented in another work of the authors [5]. However, the limited capacity of landfills for final solid wastes from biogas production plant prompts intensive research work related to experimental verification of various chemical, biochemical, environmental engineering or material engineering concepts. It indicates potential directions of useful development of these, relatively large in global scale, material streams. Therefore, the proposed, innovatively integrated process of gradual multi-stage conversion of a specific type of waste-sludge from conventional municipal wastewater treatment (alternatively agricultural waste, biomass, etc.) consists first in the optimal use of bioprocesses for the production of biogas. Then, further thermal conversion of residues is used to obtain innovative and at the same time on purpose-functionalized and therefore valuable combustion by-products. These can further be used in agriculture as, for example, widespread innovative, cheap, and functional adsorbents.

In the presented work, the authors’ attention was focused on the technological aspects of the conversion of sludge obtained in the process of municipal wastewater treatment, including methane fermentation. The choice of the research object resulted from the analysis of the problem [5], both ecological and logistic, directly related to its continuous, relatively stable production throughout the year (no clear seasonal fluctuations). 

It should be emphasized, that the presented innovative technological concept allows for effective and safe, two-step management of the solid residues formed after biotechnological processes related to biogas production. It is related to the advantageous reduction of uncontrolled emission of methane and carbon dioxide to the atmosphere, sterilization, and also relatively fast biotechnological stabilization of the post-process residues.

The necessity of technological modification of the solid anaerobic fermentation product is also closely related to the possibility of its use in agriculture. Despite the initial content of NPK compounds in the digestate sludge, due to the chemical composition and specificity of the preceding fermentation process, it cannot be stored for a long time. However, this is the basic criterion of its suitability in agriculture due to the established vegetation cycles of the crops. By using high-temperature combustion processes, it is possible to obtain both sterilization (the previous bioprocess was carried out with the use of different microorganisms) and simultaneously some physicochemical modification with intentionally introduced nanostructured additives (kaolinite, halloysite). The resulting ash-based structures allow first a gradual, slow release of naturally occurring nutrients to the soil (rhizosphere). Then, the depleted ash can be innovatively, instantly reused without any need for costly physicochemical and/or thermal regeneration as a convenient inert carrier for the cyclic introduction and release of nutrients. It may result through cyclic processes of adsorption and desorption of these substances and microelements (as a permanent, integral element of soil structure).

An important utilitarian, but equally innovative aspect is also the simultaneous introduction of specific ash structures into the soil, in particular those of the resulting structures formed in the combustion process of sludge with octahedral and tetrahedral inorganic additives. These structures play an important role in the cyclic processes of adsorption and desorption of nutrients, enabling their slow, systematic release. Thus, as a kind of buffering system, they effectively prevent disadvantageous eutrophication phenomena related to the uncontrolled, too fast introduction of large loads of nutrients into the environment over short periods of time. This especially concerns nitrogen and phosphorus from mineral fertilizers, periodically introduced into farmlands and unintentionally leached away as a result of acid rains). It should be also emphasized that the idea of the authors is based on the currently UN-recommended strategy (e.g., of the COP21 conference) to significantly reduce the phenomena of uncontrolled methane emission to the biosphere. It may be done through a versatile and innovative, effective technological use of all by-products of process-integrated methane fermentation with municipal wastewater treatment. Moreover, additional energetic utilization of solid residues (after their previous drying with the use of heat from the combustion of methane obtained in this biogas installation) can be advantageous. An integral part of this CE-based concept was optimization of the operating conditions of an integrated anaerobic digestion system with municipal wastewater treatment with the use of artificial intelligence. It was the subject of another, prior related authors’ work [5]. The presented approach, indirectly, also reduces the negative impact of greenhouse gases on the biosphere by qualitatively modifying the emissions instead of more harmful methane produced in an uncontrolled manner during the storage of solid post-fermentation residues, carbon dioxide from controlled combustion processes enters the atmosphere.

One of the important elements of the proposed innovative multi-stage concept, presented in detail in this study, is the combustion of dewatered and dried post-digestion sludge with the use of nanostructured, environmentally inert aluminosilicate species such as halloysite and kaolinite to produce advantageously functionalized inorganic solid residues ashes with adsorption properties. This approach is necessary for rational resource management and responsible sustainable waste management. At the same time, it allows to avoid unfavorable environmental effects derived from unstable accumulation of nutrients. In addition, the use of combustion allows for the sterilization elimination of harmful substances, e.g., dissolved residues of various drugs contained in municipal sewage, having a potential complex impact on the physiological assimilation of nutrients by agricultural crops.

The current trends in the management of this type of waste, in line with Circular Economy principles, indicate the desirability of designing some kind of "cascade system". In such systems the post-process residues from the previous installation can be directly utilized as valuable substance substrates in the following process stages. Such concepts, consistent with the idea of sustainable development, must usually be verified experimentally to identify optimal conditions in both subsequent processes for their full integration towards the effective sequential processing of the discussed input material. 

The paper presents an innovative, multi-stage chemical concept developed by the authors, referring to the Circular Economy assumptions, based on the use of post-process sludge from wastewater treatment and biogas production technology for combustion. In turn, the ash generated in the combustion process can, according to the authors’ concept, be reused again as an element of stabilization of soil fertilization. For the purpose of verification of this innovative concept, studies on ash desorption directly after combustion and targeted re-adsorption of selected components of mineral fertilizers important for ensuring proper growth conditions for crops were carried out. As a recycled material, such a sorbent will be characterized by low production costs, broad availability, and the possibility of widespread use in agriculture. 

Due to the potentially complex composition of the wastewater supplied to the treatment plant, resulting from the specific location of the treatment technological line (wastewater from industrial plants, municipal wastewater, etc.) and the differentiated treatment technologies used, related to the introduction of various new, secondary chemicals into the mixtures (oxidizing or reducing agents, substrates of complex reactions, species for disinfecting, for pH correction and the like), the solid phase resulting from these purification processes as a whole represents a complex system. Its complexity demonstrates itself in terms of structure, chemical, and phase composition. Unlike industrial and agricultural wastewater, showing a relative stability of composition over longer periods of time, municipal wastewater demonstrates different behavior. Sometimes it is characterized by significant, stochastic fluctuations in the concentration of individual compounds and ions, thus it is particularly difficult to predict its composition and future concentration(s) trends. Thus, the resulting solid or semi-liquid post-technological residues, required for periodic or continuous removal from the technological system, may show quite large variability of composition. This aspect ultimately has a decisive influence on their physicochemical properties determining the final application areas.

Sewage sludge arising during complex technological processes of treating various types of municipal wastewater is a subject to continuous accumulation and thus constitute an increasingly important, potential problem for the natural environment (biosphere). New technological ways of their safe management are still being sought. In particular, it is related to their possible return to the economy in a more or less processed form, in accordance with Circular Economy principles and the standards of sustainable development. One of the promising directions for the use of this type of by-product is combustion in the energy sector, as well as other methods based on thermochemical conversion processes such as pyrolysis or gasification. Direct combustion or co-incineration along with a properly selected combination of other fuels (coal, biomass, etc.) may also be applied in energy generation installations [6]. Apart from the possibility of obtaining a significant reduction of the initial volume of sewage sludge and the elimination, or at least minimization of the production of odor compounds [7], there is still the problem of purposeful management of the residues after the combustion process. Due to the complex chemical composition, sewage sludge combustion requires accounting for many other technological limitations related to, among others, ensuring stable operation of the plant, reducing the emission of potential secondary pollutants, and operating safety of the overall technological system. In this respect, particular attention should be paid to the relatively high content of heavy metals fractions, effect of various organic compounds and their combinations (pharmaceuticals residues or their decomposition products) and all other compounds with toxic properties identified in sewage sludge. In the case of sewage sludge from municipal wastewater treatment, the frequently observed qualitative (composition) and quantitative (mass or volume flux) fluctuations, leading to the potential temporary occurrence of more environmentally unfavorable (toxic) combinations of pollutants, is also important. Also of significant importance in such time-varying systems is the possibility of more environmentally unfavorable expression of certain specific pollutants due to the specific combination of other “background” wastewater components, causing stronger expression of these pollutants.

Current directions of sewage sludge management, due to technical and economic conditions, include, in addition to anaerobic digestion analyzed by the authors in another work [5], other processes related to direct energy production in combustion or co-combustion, as well as in other technologies based on high temperature conversion [8]. In the literature, one can find descriptions of sewage sludge combustion with the use of various technical solutions. The reports cover combustion in a circulating fluidized bed [9,10], bubbling fluid bed unit [11], or using the grate furnaces in stoker-fired boilers technique [12,13]. However, the authors of the present work propose an innovative integration of both approaches together; first, anaerobic digestion of the sludge, followed by drying and combustion of solid digestate (with or without additives).

Ash from incineration or co-incineration of sewage sludge (as digestate) contains relatively low amounts of chlorine compared to, for example, some types of waste biomass, straw, or the increasingly used refuse-derived fuel (RDF). For this reason, sewage sludge combustion is not related to the occurrence of a high risk of high-temperature corrosion in the combustion chamber [14]. Moreover, this ash does not show significant slagging and sintering tendencies, as well as relatively high values of ash fusion temperatures (AFT) [15,16]. Under industrial conditions, during the co-incineration of waste wood with sewage sludge, some positive trends were also observed. These were mainly related to a significant reduction in the corrosion effects compared to the effects related to the combustion of wood alone [17].

The research on ash generated in the sewage sludge combustion or co-incineration processes presented in the literature shows that the main direction of its management is primarily the possibility of phosphorus recovery and its direct use for the production of fertilizers. However, the possibilities of using it for this purpose depend mainly on the chemical composition of the ash. These are also strictly regulated by law mainly due to the minimum content of phosphorus and current standards for heavy metals concentration [18]. Attempts to extract phosphorus from sewage sludge and its precipitation in the form of struvite are also presented [19].

Nevertheless, in other publications, attention was drawn to the main problem that needs to be solved, namely, the relatively high concentration levels of metals. This results from the accumulation of pollutants in sewage sludge in a complex, multi-stage technological processes of wastewater treatment. For example, in [20] the concentrations of some tested metals (cadmium, mercury, lead, zinc) were within the acceptable standards, but the concentrations of others (copper, chromium, nickel) exceeded the acceptable limits, which was a significant technological problem. Meeting the standards made it possible to admit the ash to the technological process of the production of mineral fertilizers [21]. For this purpose, the speciation of phosphorus contained in ashes produced during the sewage sludge co-combustion process was also verified with selected types of biomass. In particular, bioavailability from the point of view of the target application in agriculture was identified [22].

Another important application trend of the sewage sludge combustion ash in question is its use as an additive in the concrete production process. Due to the lack of an appropriate standard for ashes other than coal-derived ones, its compliance with the available standard for ashes from coal combustion [23] was positively verified only. Ash can be added to cement and concrete in its raw form or after grinding [24] (initial acid-based leaching for the recovery of phosphorus compounds may reduce its usefulness [25]). The ash from the incineration of the discussed type of sludge can replace even 10% of cement, but on the other hand, it can modify its characteristics. In particular, it refers to the values of parameters such as volume deformation or early age cracking risk [26]. In another study, various shares of ash from sewage sludge combustion in the range of 5–25% by mass were verified, confirming acceptable values of such operational parameters of the product as, for example, compressive strength of concrete [27].

Another trend noticeable in the available literature, directly related to the practical use of ashes from a combination of combustion, gasification, and pyrolysis processes using sewage sludge as a fuel, is their use in sorption processes. An important aspect is both the low price of the adsorbent and the possibility of managing this type of ash in line with Circular Economy guidelines. Examples of the adsorption abilities towards elemental mercury from flue gas mixtures [28], H_2_S [29,30], nickel ions from wastewater [31], Cu(II) ions [32], Cd, and Pb [33], radioactive cesium (Cs) [34] are available.

Parameters such as polarity, specific surface area developed, combination and concentration of surface functional groups, as well as aromatization degree together with graphitization degree resulting directly from their intrinsic chemical composition [32] may play a key role in adsorption processes. The review of potential applications of sewage sludge ashes in the processes of adsorptive removal of pollutants from wastewater and some pharmaceuticals is presented in the reviews [35,36].

This work presents the results of innovative experimental research that are part of a broader research study including the original, optimal integration [5] of subsequent processes related to municipal wastewater treatment in a technological system integrated with biogas production, followed by combustion of the obtained post-digestion sewage sludge and effective, innovative use of the resulting ashes as the carriers of nutrients in agricultural applications. Thus, such innovative technical process integration is fully compliant with the Circular Economy assumptions and the principles of sustainable development. Sewage sludge represents here the sludge formed in the process of municipal wastewater treatment after its initial use in the integrated anaerobic digestion process focused on biogas production [5]. 

The presented innovative technological “core concept” may have, however, many potential variants. Depending on the structure of the chemical composition of the carrier, it can be used both in the cultivation of energy crops, flowers, shrubs, and under more stringent sanitary standards and restrictions in the cultivation of plants used for the production of food and feed products.

## 2. Materials and Methods

For the purpose of the research on innovative multi-stage Circular Economy-based technology, especially for the described in this work its “combustion-sorption” part, samples of sludge from anaerobic digestion chambers used for the production of biogas were collected at the integrated technological line located at the sewage treatment plant in Rybnik-Orzepowice, Poland (implemented quality management system compliant with the requirements of PN-EN ISO/IEC 17025: 2018-02, the Laboratory Department obtained the Accreditation Certificate No. AB 1775). A detailed model analysis of the operation of a wastewater treatment plant, technologically integrated with a biogas production system, has been presented in another work of the authors [5]. In the analyzed technological facility, biogas production takes place with the use of two fermentation chambers (bioreactors), each with a working volume of 2500 m^3^. The process of biogas production runs under the conditions of anaerobic mesophilic fermentation (about 37 °C), which enables the achievement of methane concentrations in biogas up to 65%. Raw sludge from wastewater treatment is used as a substrate in the integrated anaerobic digestion process. This raw sludge is separated in the primary sedimentation tanks of the sewage treatment plant. It is then thickened by gravity in an appropriate gravity thickener until reaching a concentration of 4.5–5.0% dry weight. The obtained excess sludge is then concentrated in the mechanical thickener until it reaches the level of 5.0%. It also contains fat-like compounds trapped in the structure of the aerated sand bed.

After the anaerobic fermentation process in the fermentation chambers of the biogas production system, the post-digestion residues in the form of sludge are then transported to the storage tank and dewatered with the use of an appropriate filter press. In 2021, the total volume of sludge supplied to bioreactors anaerobic digestion chambers was 36,378 m^3^ (monthly fluctuations—crude sludge: minimum 2594 m^3^—maximum 3390 m^3^, excess sludge: minimum 1488 m^3^—maximum 2136 m^3^, fats: minimum 7 m^3^—maximum 44.1 m^3^). The mass of sludge produced after the dewatering processes with the use of a filter press (dewatered sludge) in the analyzed technological facility was in total 8280 Mg/year (in monthly analysis: minimum 520 Mg, maximum 833 Mg).

For the ongoing control of the anaerobic fermentation in bioreactors, pH, VOC concentration, dry matter value (%), mineral fraction (%), and volatile matter fraction (%) are periodically monitored and registered (SCADA). After dewatering in the filter press, the sludge is hygienized with quicklime. The chemical composition of dewatered post-digestion sludge (for 2021) is presented in Table 1.

The samples of post-digestion sewage sludge were received in a form of a wet slurry. Their initial moisture content was determined by weight method according to PN-EN ISO 18134-2:2017-03 and the initial ash content by oven method according to PN-EN ISO 18122:2016-0, with the results of 81.7 wt% and 7.5 wt%, respectively. Due to high moisture content, the sludge was pre-dried at an ambient temperature and then placed for 12 h into an electric dryer at a temperature of 115 °C for simultaneous drying and sterilization. Such dried and sterilized sewage sludge was taken for further investigation and analyses.

The dried sludge was grounded in a laboratory knife mill and divided into three samples, one without any additives (reference sample) and two samples doped with the aluminosilicate fuel additives halloysite and kaolinite. The additive dose was established to be 2 wt%, based on the authors’ previous research experience [37,38]. Each additive was admixed into the post-digestion sewage sludge before incineration. After the preparation of sludge-additive blends, all samples were incinerated in an electric muffle furnace according to the same procedure and under the same conditions. A small batch of sludge was placed in a ceramic crucible, heated up, and incinerated in an air atmosphere and constant temperature zone of 550 °C, so that a low-temperature, chemically stable ash was obtained. The temperature of 550 °C was chosen according to the standard method for the determination of ash content of all solid biofuels PN-EN ISO 18122:2016-0. As a result, three ash samples were obtained for further investigation, i.e., post-digestion sewage sludge-derived ash without any additives (A1), ash from post-digestion sewage sludge incineration with kaolinite (A2), and ash from post-digestion sewage sludge incineration with halloysite (A3).

Proximate and elemental analysis of post-digestion sewage sludge was done to identify its fuel characteristics. For the ash, the oxide ash composition was determined together with ash fusion temperatures (AFT) as it is one of the most common methods for ash characterization.

Fuel analysis was done according to European standards: moisture content PN-EN ISO 18134-2:2017-03, ash content PN-EN ISO 18122:2016-01, Higher Calorific Value (HHV) and Lower Calorific Value (LHV) PN-EN ISO 18125:2017-07, chlorine, and sulfur contents by IC (Ion Chromatography) method according to PN-EN ISO 16994:2016-10, carbon, hydrogen and nitrogen contents by IR (Infrared) automatic analyzer according to PN-EN ISO 16948:2015-07. Chemical composition of ash was determined using ICP-OES (Inductively Coupled Plasma-Optical Emission Spectrometry). Ash fusion temperatures were determined by the microscope-photographic method according to standard CEN/TS 15370-1:2007. The tests were done assuming a maximum temperature of 1500 °C and both oxidizing and reducing conditions. Test procedures covered identification and recording of initial deformation temperature (IDT), softening temperature (ST), hemisphere temperature (HT), as well as the flow temperature (FT). These were done considering the specific, characteristic shapes of the ash cylinders detected by a camera system driven and controlled by a PC. 

Proximate analysis, elemental analysis, and heating values of post-digestion sewage sludge are presented in Table 2. The ash content was found to be significantly high (58.3%). The calorific value can be considered relatively high for this type of fuel. Contrary, chlorine content is considered to be relatively low (0.089%). 

The chemical composition of the ashes, together with ash fusion temperatures is summarized in Table 3. The post-digestion sewage sludge-derived ash is characterized by a considerable content of alkaline compounds such as calcium (29.49%) and potassium (1.87%). These can be responsible for slagging and fouling during the combustion process. The ash also contains a high amount of silica (30.20%) and phosphorus (14.72%), typical for this type of waste [21]. Silica, together with chlorine, sodium, potassium, and other alkali metals leads to the formation of eutectics with low melting point temperatures [39,40]. Nevertheless, the ash fusion temperatures of investigated ash can be considered high, which is likely to be the result of relatively low chlorine content (0.089%). The characteristics of investigated sewage sludge ash indicates its low-to-moderate tendency for deposition, slagging, fouling, and high-temperature corrosion since these issues are strictly dependent on AFTs and chlorine content [41]. The addition of halloysite and kaolinite is likely to further improve the combustion properties by bonding sodium and potassium, mainly in the form of chlorides with higher melting points [42]. In practical terms, the aluminosilicate additives may reduce slagging and fouling of boiler heating surfaces, as well as deteriorate the agglomeration processes in fluidized beds. Moreover, such additives reduce the concentration of KCl and NaCl present in the ash deposits, thus the rate of high-temperature corrosion is expected to decrease [43]. As indicated, the additive dose of 2 wt.% was determined based on the authors’ previous experience with various biomass fuels.

The solutions for the research on the adsorption processes, containing the tested ions, were prepared by adding the original standard solutions of: NO_3_^−^, K^+^, PO_4_^3−^ and Mg^2+^, Na^+^, Cl^−^, SO_4_^2−^ (with a concentration of 1000 mg/dm^3^) from Merck to deionized water to obtain appropriate concentrations. The identification of the ions concentrations in the sample solutions was done with the appropriate Merck and Hach reagents (NO_3_^−^, K^+^, Cl^−^) and cuvette tests (PO_4_^3−^, SO_4_^2−^, Na^+^):

− NO_3_^−^ ions, no. 09713 by Merck,
− K^+^ ions, no. 00615 by Merck,
− Na^+^ ions, No. 00885 by Merck,
− Cl^−^ ions, no. 14897 by Merck,
− PO_4_^3−^ ions, Hach No. LCK350,
− SO_4_^2−^ ions, Hach No. LCK153.

Instrumental measurements of ion concentrations in the solutions (before and after the sorption processes) were done using the Spectroquant Pharo100 by Merck (Darmstadt, Germany) and the DR 5000 by Hach Lange (Berlin, Germany).

Before starting the studies of the adsorption isotherms of selected compounds on the adsorption surfaces of the prepared ash samples, the solvent extraction of the three groups of adsorption materials was carried out with the use of distilled water. It was aimed at the possibly complete removal of nutrients naturally present in the ash (in particular, these were: NO_3_^−^, K^+^, Na^+^, PO_4_^3−^, SO_4_^2−^, Cl^−^). Thus, experimental determination of the “pure” adsorption capacity of only the remaining inert structure (matrix) in respect to the selected ions was possible. This may be of significant importance in practical applications that require cyclical processes of adsorption and desorption (slow release of some nutrients in the “spontaneous extraction process” to the natural environment, e.g., soil). For this purpose, the process of solvent extraction of all ash samples (A1, A2, and A3—see Table 3) was carried out with the use of distilled water. After each batch extraction process, the obtained solution was replaced with fresh distilled water. The applied procedure corresponded to the technological system, including an initial 25 stages of cross-current extraction carried out in a batch mode, e.g., in an extraction battery. After the last extraction stage, an instrumental analysis of the concentration of individual ions in the supernatant liquid (after filtration) was done (separately for each of the three ashes—A1, A2, and A3). The results were as follows:

- Cl^−^: A1–1.8 mg/dm^3^, A2–(1.7–1.9 mg/dm^3^), A3– (2.3–2.4 mg/dm^3^),
- NO_3_^−^: A1–(8.7–9.5 mg/dm^3^), A2–(7.9–8.0 mg/dm^3^), A3–(8.2–8.3 mg/dm^3^),
- K^+^: A1 < 30 mg/dm^3^, A2 < 30 mg/dm^3^, A3 < 30 mg/dm^3^,
- Na^+^: A1–0, A2–0, A3–0,
- SO_4_^2^^−^: A1–0, A2–(7.93–8.04 mg/dm^3^), A3–0,
- PO_4_^3^^−^: A1–(1.76–1.79 mg/dm^3^), A2–(1.59–1.61 mg/dm^3^), A3–(1.63–1.68 mg/dm^3^).

The obtained “depleted” sludge samples, representing wet A1, A2, and A3, respectively, were dried for a period of 24 h using a laboratory dryer FD-S 056 by Binder (Tuttlingen, Germany). The temperature of the drying process was 50 °C with air circulation.

To carry out the experimental identification of the equilibrium conditions for the adsorption of selected components in the tested physicochemical systems, the samples (6 g each mass) of the dry, “depleted” adsorbent substance: P1, P2, and P3 were prepared. Then, the prepared masses of adsorbents were brought into contact with aqueous solutions (based on distilled water) containing combinations of selected ions, each solution having a constant volume of 35 cm^3^. For the adsorption equilibrium (isotherm) tests, five aqueous solutions (S1–S5) of gradually increasing concentrations of six selected ions (NO_3_^−^, K^+^, Na^+^, PO_4_^3−^, SO_4_^2−^, Cl^−^) were used. 

Then, these were shaken on a shaker (apparatus Sk-0330-Pro by ChemLand, Stargard, Poland) for 8 h, and left for another 15 h to rest for undisturbed gravitational sedimentation.

For each sample of the solution, due to the key importance of the result of the concentration value for the correct determination of the equilibrium distribution of the adsorbate between the solid adsorbent and the solution, the measurement was performed three times.

The three direct concentration measurement results followed by arithmetic mean concentration value (mean was used in the models of adsorption isotherms) of each analyzed component in these solutions (S1–S5). Composition of post-adsorption solutions (in equilibrium) and adsorption results on solid phase are also presented.

The adsorbate mass per unit mass of the adsorbent in equilibrium, *q*_e_, was determined according to the Equation (1):

(1) $$q_{e}=\frac{\left(C_{0}-C_{e}\right)V}{m}$$

where *C*_0_—initial concentration of the substance adsorbed from the solution (mg/dm^3^), *C*_e_—equilibrium concentration of the substance adsorbed from the solution (mg/dm^3^), *m*—adsorbent mass (g), *V*—volume of the solution in each analyzed sample (dm^3^).

For the theoretical interpretation of the results, linearized equations of adsorption isotherms were used, namely, models of:

Langmuir (2) [44]:

(2) $$\frac{C_{e}}{q_{e}}=\frac{C_{e}}{q_{m}}+\frac{1}{K_{L}q_{m}}$$

and Freundlich (3) [44]:

(3) $$\ln\left(q_{e}\right)=\ln\left(K_{F}\right)+\frac{1}{n}\ln\left(C_{e}\right)$$

In Equations (2) and (3), *K*_L_ represents the constant of the Langmuir adsorption isotherm (2) (dm^3^/mg), *q*_m_ interpreted as the maximum possible adsorbate mass per unit mass of the adsorbent (which in the light of Langmuir’s theory expresses the maximum covering of surface area by a monolayer adsorbed substance, mg/g), while *K*_F_ and *n* are the constant and exponent of the Freundlich isotherm model Equation (3).

Considering the limited measurement data, the possible simplest two parameter models of adsorption isotherms were deliberately selected.

Additional model calculations were performed using Temkin adsorption isotherm model (4) [45]:

(4) $$q_{e}=B_{T}\;ln\left(A_{T}C_{e}\right)$$

and a simplified model of Jovanović adsorption isotherm (5) [46]:

(5) $$q_{e}=q_{J}\;\left[1-\exp\left(-K_{J}\;C_{e}\right)\right]$$

Both equations of adsorption isotherms represent two-parameter models. In the Temkin isotherm model (4), it is assumed that the heat of adsorption for all molecules in the layer will decrease linearly with increasing coverage of the available adsorbent surface. In the Temkin isotherm model (4), the *A*_T_ parameter is called the Temkin isotherm constant (dm^3^/mg), while the second parameter *B*_T_ is called Temkin isotherm energy constant (mg/g). On the other hand, in the Jovanović isotherm model (5), the *q*_J_ (mg/g) parameter expresses the theoretically predicted maximum (asymptotic according to the model) adsorption capacity of the surface of the tested material, while *K*_J_ is the equilibrium constant of the model (dm^3^/mg).

## 3. Results

### 3.1. Adsorption Isotherms

Analytical results concerning the possibility of adsorption of individual species from aqueous multi-component solutions using the three variants of the innovative adsorbent are presented in Section 3.1.1, Section 3.1.2, Section 3.1.3, Section 3.1.4, Section 3.1.5 and Section 3.1.6.

#### 3.1.1. Adsorption Characteristics of the Post-Digestion Sludge Ash-Derived Sorbents in Respect to NO_3_^−^ Ions

The experimental data concerning adsorption of NO_3_^−^ ions on three types of waste post-digestion sludge ash-derived adsorbents is presented in Table 4.

Based on the data presented in Table 4, it can be seen, that in the case of adsorptive removal of nitrate(V) NO_3_^−^ ions from aqueous solutions, the application of ash from the combustion of post-digestion sludge from biogas production as an adsorbent without any additives gives the best process results. Depending on the value of the initial concentration of nitrate(V) ions in the contacted liquid phase, from about 11 to about 13% of their removal is confirmed experimentally. It is also the indirectly expressed adsorption capacity of the analyzed material in respect to nitrate(V) ions. In the case of the material obtained during the combustion of the digestate matter without the use of any additives, there is no clear quality trend between the initial concentration of nitrate(V) ions in the solution and the adsorption effect on the ash-based sorbent. Regardless of the concentration of the adsorbate in the solution, the average efficiency of its removal from the solution is about 12%.

The combustion of the post-fermentation sludge together with kaolinite causes a noticeable reduction in the adsorption capacity of nitrate(V) ions of the thus obtained material based on the more complex in composition waste ash. Increasing the concentration of NO_3_^−^ from about 58 to 173 mg/dm^3^ resulted in a clear, gradual reduction in the adsorption capacity (from 9.82 to 3.66%) of the sorption material, representing this time a structure produced in the high-temperature process of burning sludge with kaolinite. Similarly, it is possible to compare both analyzed process systems with the ash resulting from the combustion of post-fermentation sludge with the addition of halloysite. A similar, decreasing trend is visible. But it is noticeable only between solutions S2 and S3. In the case of both identical solutions S2 and S3, higher adsorption effects (5.42 and 7.13%, 3.66 and 4.05%, respectively) are noticeable when halloysite is used as an additive in the combustion process.

Theoretical analysis of the established concentration values of the equilibrium distribution of NO_3_^−^ ions between the solid phase (adsorbed fraction) and the liquid phase (the remaining concentration in the contacted solution) allows approximating and suggesting the mechanism of the sorption process. However, due to the limited measurement data (see Table 4), the presented considerations should be interpreted only as a preliminary and rough approximation of the potential sorption mechanism.

In the case of ash obtained from the combustion of post-fermentation sludge without any additives potentially affecting the combustion process, it was not possible to obtain theoretically meaningful parameters of the Langmuir isotherm model—Equation (2). Alternative application of Equation (3)—the Freundlich adsorption isotherm, made it possible to obtain a relatively high *R*^2^ = 0.989 (*K*_F_ = 0.000505, *n* = 0.913), indicating, based on the theoretical assumptions of the model, the imperfectness of the analyzed adsorption process. Moreover, the Freundlich model suggests in this case a heterogeneous morphology of the available adsorption surface obtained from the combustion of sludge deposits. Additionally, in this case it can be assumed, that the multilayer adsorption phenomena of nitrate(V) ions on the adsorbent surface may prevail.

The value of *n* < 1 suggests, first of all, relatively weak binding interactions between the adsorption surface and the adsorbate, and thus unfavorable conditions of the adsorption process in the thus defined physicochemical system.

However, for the ash obtained from the combustion of post-fermentation sludge with the addition of kaolinite, after verifying the possibility of describing the measurement data using the Langmuir isotherm model—Equation (2) (*R*^2^ = 0.996, *q*_m_ = 0.039 mg/g, *K*_L_ = 0.1404 dm^3^/mg) and Freundlich isotherm model—Equation (3) (*R*^2^ = 0.730, *K*_F_ = 0.0224, *n* = 9.66), the Langmuir model (2), turned out to be much better.

The introduction of halloysite to the combustion system of the post-fermentation sludge instead of kaolinite resulted in the formation of a qualitatively different surface structure. It demonstrated some affinity in relation to nitrate(V) ions as a result of thermochemical reactions. Regression analysis using both isotherm models (2) and (3) gave the following results–Langmuir model: *R*^2^ = 0.722, *q*_m_ = 0.0627 mg/g, *K*_L_ = 0.0147 dm^3^/mg, Freundlich model: *R*^2^ = 0.657, *K*_F_ = 0.003, *n* = 1.84.

Higher than 1 values of the *n* coefficient in the latter two cases (the use in the combustion process the additives in the form of kaolinite and halloysite) suggest that the main process mechanism in these two cases is physical adsorption. These data also indicate favorable conditions for this type of adsorption process. The approximately five-times higher value of the *n* coefficient obtained in the case of using kaolinite in the combustion process indicates the possibility of the purposeful addition of kaolinite for the targeted production of adsorption surfaces with a specific selectivity towards nitrate(V) ions.

It should be emphasized here, that due to the limited number of measurement data and relatively small values of *R*^2^, the obtained data should be treated only as preliminary, rough approximate values.

Regarding the possibility of adsorption of nitrate(V) ions on ash without nanostructural additives, the Temkin isotherm (4) gives the calculated parameter values as *B*_T_ = 0.1034 mg/g, *A*_T_ = 0.0253 dm^3^/mg (*R*^2^ = 0.912). The presence of kaolinite in the conditions of sludge combustion is represented by the values *B*_T_ = 0.00364 mg/g and *A*_T_ = 200.87 dm^3^/mg (for *R*^2^ = 0.718). For ash with halloysite, the Temkin isotherm model (4) predicts the *B*_T_ parameter value of 0.0177 mg/g, while the *A*_T_ value is 0.0872 dm^3^/mg (for *R*^2^ = 0.574).

In the case of ash without nanostructural additives, the Jovanović isotherm model (5) predicts the maximum adsorption capacity of the material in relation to nitrate(V) ions *q*_J_ = 2.904 mg/g (*K*_J_ = 0.0002989 dm^3^/mg, *R*^2^ = 0.973). According to the model, the introduction of kaolinite in the combustion process causes a reduction in the value of *q*_J_ = 0.0377 mg/g, *K*_J_ = 0.0414 dm^3^/mg, *R*^2^ = 0.915). The presence of halloysite under combustion conditions increases the value of the maximum adsorption capacity up to *q*_J_ = 0.0493 mg/g (*K*_J_ = 0.016 dm^3^/mg, *R*^2^ = 0.636). However, this value is still lower than the maximum adsorption capacity of ash without additives predicted by the same model. Nevertheless, the relatively low values of the *R*^2^ coefficient of all three Jovanović isotherm models for A1–A3 allow for only limited conclusions to be drawn.

#### 3.1.2. Adsorption Characteristics of the Post-Digestion Sludge Ash-Derived Sorbents in Respect to K^+^ Ions

The experimental data concerning adsorption of K^+^ ions on three types of waste post-digestion sludge ash-derived adsorbents is presented in Table 5.

Analyzing the adsorption potential of ash from the combustion of post-digestion sludge in relation to potassium K^+^ ions in aqueous solutions, their gradual decrease, along with the concentration of adsorbate in the analyzed aqueous solutions, can be noticed. The decrease in the adsorption capacity of the material with the increase in the concentration of the adsorbate in the solution is consistent with the nature of the adsorption process. In adsorption the highest process efficiency is usually observed in the case of diluted solutions (for such solutions, the adsorption process is usually recommended).

However, a significant reduction in the adsorption capacity of the tested material between solutions S3 and S4 (Table 5) and almost identical removal of the potassium ions from solutions S4 and S5 (Table 5) can be noticed. A clear reduction in the adsorption capacity towards the K^+^ ions is also visible in the case of ash derived from the combustion of dried post-digestion sludge with the addition of kaolinite. However, in this case, the decrease in adsorption capacity is slower and is noticeable in a wider range of S2–S5 concentrations (Table 5): 20% → 11.81% → 9.20% → 0.27%. Thus, the advantageous effect of the addition of kaolinite in the combustion process on the resulting surface properties of the sorption material is clearly visible, increasing its adsorption efficiency in relation to K^+^ ions in the range of their higher concentrations. 

On the other hand, the addition of nanostructured halloysite during the combustion of the dried sludge from anaerobic fermentation causes a noticeable, however moderate, increase in the K^+^ adsorption capacity of the obtained ash-based material in the range of higher solution concentrations. For comparison, the values corresponding to S3 solutions: 16.54% and 11.81%, S4: 13.19% and 9.20% or 1.53% as well as S5: 1.09% and 0.27% and 1.09%. This is a favorable trend, effectively offsetting the generally lower adsorption capacity in the range of increasing concentrations of adsorbate in aqueous solutions.

As a result of the theoretical analysis using the adsorption isotherm models in the case of ash obtained in the combustion process without additives, no results with a justified physical sense were obtained. For the adsorbent represented by ash formed in the combustion conditions with the addition of kaolinite, the possibility of using the Freundlich isotherm model was identified (physical sense of the model parameter values), despite the relatively low value of the *R*^2^ coefficient (*R*^2^ = 0.902, *K*_F_ = 0.0483, *n* = 23.855).

The addition of halloysite in the combustion conditions of post-digestion sludge resulted in the formation of adsorptive structures, representing mixture of octa and tetrahedrons together with structures remaining after elimination of organic coal in the processes of gasification and thermal conversion. For these the following values of the Freundlich model parameters were obtained: *R*^2^ = 0.912, *K*_F_ = 0.00536, *n* = 1.62. In the case of the ash obtained in the presence of halloysite, the Langmuir isotherm model (*R*^2^ = 0.850, *q*_m_ = 0.1694 mg/g, *K*_L_ = 0.0111 dm^3^/mg) also gives comparable results.

The higher value of the *K*_F_ constant corresponding to the adsorbent thermosynthesis in the conditions of kaolinite co-presence (*K*_F_ = 0.0483) than the co-presence of halloysite (*K*_F_ = 0.00536) suggests more advantageous conditions for the affinity of K^+^ ions to the prepared in this way adsorption structure. Both values of the parameter *n* are higher than 1 (for kaolinite *n* = 23.855, for halloysite *n* = 1.62), which, on the basis of theory, suggests the existence (or rather, dominance) of the physical adsorption mechanism. It also indicates favorable conditions for the adsorption of potassium ions on such adsorbent, significantly better for kaolinite (clearly reflected in the data presented in Table 5).

The use of the Temkin isotherm Equation (4) to model the course of the obtained experimental data resulted in the following sets of parameters. For the ash obtained without additives *B*_T_ = 0.0114 mg/g, *A*_T_ = 3.794 dm^3^/mg (however, unacceptable *R*^2^ = 0.015), ash produced in the presence of kaolinite *B*_T_ = 0.003 mg/g, *A*_T_ = 4346978 dm^3^/mg (*R*^2^ = 0.852), and for the ash produced in the presence of halloysite *B*_T_ = 0.0416 mg/g, *A*_T_ = 0.086 dm^3^/mg (*R*^2^ = 0.913). Large fluctuations in the values of both parameters of the Temkin isotherm model, as well as low *R*^2^ values make it impossible to formulate reliable conclusions about the influence of the tested additives.

For the ash without additives, the Jovanović model (5) presented an unacceptable level of *R*^2^ = 0.034, which made it impossible to further consider its predictions in the analysis (inappropriate model). Contrarily, in the case of the presence of kaolinite in the system subjected to the combustion process, the maximum adsorption capacity in respect to K^+^ ions predicted by the Jovanović model is *q*_J_ = 0.0584 mg/g (*K*_J_ = 0.0815 dm^3^/mg, *R*^2^ = 0.997). The presence of halloysite in the combustion environment causes, according to the isotherm model (5), increase in the maximum adsorption capacity up to *q*_J_ = 0.1114 mg/g (*K*_J_ = 0.0159 dm^3^/mg, relatively low *R*^2^ = 0.915). A clear increase in the value of the *R*^2^ parameter for the surface with additives (in case of both models (4) and (5)) indicates a possible alteration in the process mechanism, which is the basis for obtaining these theoretical Equations (4) and (5) of the adsorption isotherms, individually.

#### 3.1.3. Adsorption Characteristics of the Post-Digestion Sludge Ash-Derived Sorbents in Respect to Na^+^ Ions

The experimental data concerning adsorption of Na^+^ ions on three types of waste post-digestion sludge ash-derived adsorbents is presented in Table 6.

When analyzing the results (Table 6) relating to the adsorption of sodium ions, it can be noticed that the only effects of the minimum adsorption efficiency of adsorbent structures can be observed in the case of ashes resulting from the combustion of post-digestion sludge with process additives−kaolinite (2.87−3.23%) and halloysite (7.60%, a result of almost 2.35-times higher than under analogous conditions for the ash obtained by using kaolinite).

In the case of Na^+^ ions both for the ash obtained without additives and with the addition of halloysite and kaolinite, the lack of sufficient data (and thus the justified application of regression methods) makes it impossible to apply regression to determine the adsorption isotherm constants of Langmuir (2), Freundlich (3), Temkin (4), and Jovanović (5) models.

#### 3.1.4. Adsorption Characteristics of the Post-Digestion Sludge Ash-Derived Sorbents in Respect to PO_4_^3−^ Ions

The experimental data concerning adsorption of PO_4_^3−^ ions on three types of waste post-digestion sludge ash-derived adsorbents is presented in Table 7.

The measurement data presented in Table 7 show a very beneficial effect of the process additives in the form of kaolinite and halloysite for the combustion of dried post-digestion sludge. These additives not only have a positive effect on the course and parameters of the combustion process itself, but as it can be seen from the quoted data have a very positive effect on the formation of the adsorption surface within the ash-based material.

Due to measurement difficulties (multi-stage analytical procedures), reliable results in the case of ash from the combustion of post-digestion sludge without additives were obtained only for the highest concentrations of phosphate(V) ions which corresponded to solutions S4 and S5 (Table 7). The obtained adsorption values, expressed as the removal of these ions from the solution, are 1.13% (S4 solution) and 14.50% (S5 solution). These values are much lower than the identified adsorption abilities of ashes representing the burning of post-digestion sludge with kaolinite (removal of phosphate(V) ions in the range of 48.64–81.98%) or with halloysite (45.27–76.19%).

Comparing the adsorption possibilities against phosphate(V) ions for all three analyzed variants of ash from the combustion of post-digestion sludge (without additives, with kaolinite and with halloysite) in the range of the highest (S4 and S5) concentrations, it turns out that the best additive for increasing the adsorption capacity of PO_4_^3−^ ions turned out to be halloysite. When halloysite is used as an additive in the combustion process, a surface and ash structure favorable in terms of phosphates(V) adsorption are produced. This is clearly visible when comparing the adsorption efficiency for the S4 solution (76.19%, compared to 61.24% for kaolinite and only 1.13% for the technological version without any additives during combustion) and for S5 solution (70.13%, relative to 65.44% for kaolinite and 14.50% in the absence of additives that favorably modify the structure of the adsorbent during its synthesis in heterogeneous thermochemical combustion processes).

Theoretical analysis of the process with the use of adsorption isotherm equations was done. It showed that in the case of the adsorbent represented by ash from the combustion of post-digestion sludge without additives, no physically justified values of the parameters of both models–Equations (2) and (3) were obtained. Similarly, when analyzing the adsorption capacity of the ash obtained in the process conditions with the presence of halloysite. In the case of the theoretical analysis of the adsorption structure represented by the ash formed in the conditions of kaolinite co-presence, much better results were obtained using the Freundlich isotherm model approach (*R*^2^ = 0.993, *K*_F_ = 2.53 × 10^−5^, *n* = 0.4287) than Langmuir isotherm model (*R*^2^ = 0.306, *q*_m_ = 0.6342 mg/g, *K*_L_ = 0.0762 dm^3^/mg). Nevertheless, the value of *n* < 1 suggests theoretically relatively weak interactions between the thus defined adsorption surface and the adsorbate represented by phosphate(V) ions. Similarly, the relatively low value of the Freundlich model constant 2.53 × 10^−5^ indicates theoretically worse, low affinity of PO_4_^3−^ ions with regard to the discussed adsorbent.

For the Temkin adsorption isotherm model (4), reliable results were obtained only for the presence of kaolinite under combustion conditions. The values of the isotherm parameters are *B*_T_ = 1.4367 mg/g, *A*_T_ = 0.0213 dm^3^/mg (with a high value of *R*^2^ = 0.994 proving good statistical compliance of the isotherm model (4) with the obtained laboratory data).

In the case of adsorption processes of phosphate(V) ions, the Jovanović (5) isotherm model allows for obtaining certain physicochemical data only in the case of combustion with the addition of kaolinite. The relatively high value of the maximum adsorption capacity *q*_J_ = 24.3622 mg/g (*K*_J_ = 0.000395 dm^3^/mg) predicted by the model may be problematic in interpretation due to the relatively low value of the coefficient *R*^2^ = 0.732. Nevertheless, this value is at least partially justified by the experimental data confirming the high efficiency of the tested surface structures with additives in sorption of phosphate(V) ions from aqueous multicomponent solutions.

#### 3.1.5. Adsorption Characteristics of the Post-Digestion Sludge Ash-Derived Sorbents in Respect to SO_4_^2−^ Ions

The experimental data concerning adsorption of SO_4_^2−^ ions on three types of waste post-digestion sludge ash-derived adsorbents is presented in Table 8.

When analyzing the data presented in Table 8, concerning the adsorption efficiency of sulphate(VI) ions for the adsorbent represented by ash from post-digestion sludge combustion without process additives, some fluctuations can be noticed. This can be partially explained by the stochastic structures of the analyzed adsorbents. In general, it can be concluded, that the values of the adsorption efficiency fluctuate relative to the average value of ca. 10.9%.

On the other hand, in the case of the two remaining ashes, produced under the conditions of heterogeneous chemical reactions of the complex structure of the post-digestion sludge (in terms of composition, proportion, and initial spatial structure) and inorganic additives, interesting trends related to the modification of the adsorption efficiency in respect to SO_4_^2−^ ions can be noticed. In the case of the kaolinite additive, the measurement data related to the S2–S5 solutions clearly show a gradual increase in this efficiency, i.e., 1.72% → 11.62% → 17.81% → 18.23%. The highest increase in sorption efficiency is observed between the solutions S2 and S3, amounting to almost 10%, then about 6% and finally about 0.5% only. A similar situation occurs in the case of the analysis of ash generated in the combustion processes of post-digestion sludge with the addition of halloysite. However, it is clearly visible that in this case the analytically identified values of the adsorption efficiency of sulphate(VI) ions are higher for individual solutions, i.e., S2 (5.85% compared to 1.72% for kaolinite), S3 (14.19% compared to 11.62% for kaolinite), or S4 (20.53% relative to 17.81% for kaolinite). It should be added, that in the case of classical adsorption processes, their efficiency usually decreases with increasing concentration of the adsorbate in the solution; however, in both observed cases, this efficiency increases rather with increasing SO_4_^2−^ ions concentration in the S2–S4 solutions.

Theoretical analysis of the results with the use of the Langmuir (Equation (2)) and Freundlich (Equation (3)) adsorption isotherms shows that in none of the three analyzed technological variants of useful ashes the Langmuir model provides physically realistic values that can be interpreted theoretically. On the other hand, Freundlich adsorption isotherm model (3) for ash produced in the combustion environment without additives (*R*^2^ = 0.450, *K*_F_ = 1.12 × 10^−9^, *n* = 0.3547), ash produced in the combustion process with the addition of kaolinite (*R*^2^ = 0.809, *K*_F_ = 1.43 × 10^−16^, *n* = 0.1983) and with the addition of halloysite (*R*^2^ = 0.8559, *K*_F_ = 5.72 × 10^−10^, *n* = 0.3342) are problematic in interpretation due to the relatively poor values of the *R*^2^ coefficient. However, the *K*_F_ model constant and the exponent *n* values suggest more favorable conditions for adsorption of sulphate(VI) ions from aqueous solutions in the case of halloysite use in the combustion of digestate sludge. Thus, it becomes possible to rationally select and control the additives (type, concentration, possibly parameters of the combustion process, etc.) to produce the desired structure with as precisely defined application areas as possible (impact on the efficiency and selectivity of the process of adsorptive removal of selected ions from solutions).

In the case of using the Temkin isotherm model (4), it can be noticed, that for the ash obtained during combustion without nanostructural additives, the theoretically justified application of this model may be problematic due to low compliance with the experimental data (*R*^2^ = 0.603, *B*_T_ = 2.667 mg/g, *A*_T_ = 0.0011 dm^3^/mg). On the other hand, the introduction of kaolinite (*R*^2^ = 0.987, *B*_T_ = 3.8615 mg/g, *A*_T_ = 0.0011 dm^3^/mg) or halloysite (*R*^2^ = 0.931, *B*_T_ = 3.2859 mg/g, *A*_T_ = 0.0012 dm^3^/mg) causes a significant increase in the statistical compliance of the model with laboratory data. This may indicate a possible change in the process mechanism, clearly and directly related to the presence of nanostructured additives in the combustion environment, which actively contribute to the final formation of the sorption properties of the surface produced, and which can be theoretically interpreted on the basis of the assumptions of the Temkin isotherm model (4).

Calculations with the use of Jovanović (5) isotherm model show that in the case of sulphate(VI) ions, the predicted values of the maximum level of adsorbent saturation with these ions are rather overestimated and inaccurate (low *R*^2^ values). This model predicts for ash without nanostructural additives *q*_J_ = 113.56 mg/g (*K*_J_ = 0.00000778 dm^3^/mg, *R*^2^ = 0.408), for ash with kaolinite *q*_J_ = 8.755 mg/g (*K*_J_ = 0.000129 dm^3^/mg, *R*^2^ = 0.521) and for the ash with halloysite *q*_J_ = 53.37 mg/g (*K*_J_ = 0.0000223 dm^3^/mg, *R*^2^ = 0.630).

#### 3.1.6. Adsorption Characteristics of the Post-Digestion Sludge Ash-Derived Sorbents in Respect to Cl^−^ Ions

The experimental data concerning adsorption of Cl^−^ ions on three types of waste post-digestion sludge ash-derived adsorbents is presented in Table 9.

Due to the resulting analytical difficulties, the effects of chloride ions adsorption were identified only for some process systems. For ash without any additives A1 (reference ash) they were S1 and S4, for ash obtained with kaolinite co-presence A2–S1 and S2, and for the ash obtained with halloysite co-presence A3 also S1 and S2. Comparing the results for the S1 solution with relatively the lowest adsorbate concentration used, the highest (30.2%) efficiency of Cl^−^ ions adsorption was observed for the ash obtained as a result of burning post-digestion sludge without any additives (A1). It was practically comparable (31.84%) with the adsorption properties of ash A3 resulting from the combustion of post-digestion sludge with the addition of halloysite. This may prove that the addition of halloysite may positively influence only the parameters of the combustion process of the dried post-digestion sludge itself. On the other hand, its presence in the structures of the adsorbent (ash) produced as a result of thermochemical reactions seems to be rather neutral in relation to the possible change in the adsorption efficiency of chloride ions.

At the same time, it should be noted that when kaolinite was used in the combustion process, the resulting ash A2 adsorption structures showed that the adsorption capacity of chloride ions was almost by half lower than in cases A1 and A3. For the remaining analyzed solutions, with lower concentrations, much lower adsorption efficiency of the obtained post-process material A2 was observed. In the case of ash obtained without additives and with the addition of kaolinite, the observed efficiencies were 1–2%. When using halloysite in the post-digestion sludge combustion as an additive, the obtained ash demonstrated a much higher adsorption efficiency of chloride ions (9.34%) for the S2 system.

Theoretical analysis of the chloride ions adsorption using the three adsorbent structures based on combustion ash (with one selected additive, A2 or A3, or without any additives, i.e., A1) of post-digestion sludge from a biogas production installation integrated with a wastewater treatment plant (Langmuir and Freundlich model—Equations (2) and (3)) did not allow for obtaining the values of parameters having a physical sense. From this reason these could not be potentially used for a more detailed analysis of the mechanisms of the adsorption phenomena.

In the case of chloride ions, also the use of the Temkin (4), as well as Jovanović (5) adsorption isotherms did not provide reliable and consistent modelling results.

### 3.2. Scanning Electron Microscopy Analysis

**Figure 1:**
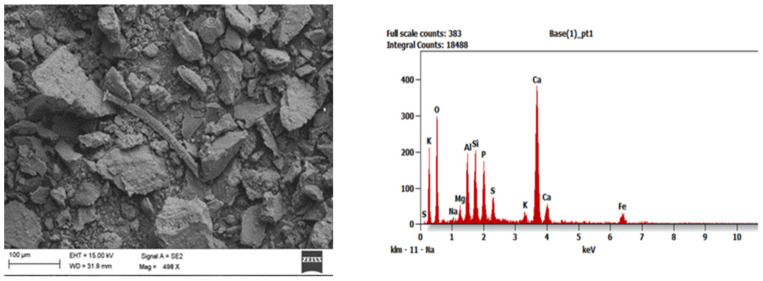
SEM image of the post-digestion sludge-based ash (combustion without additives) and energy dispersive X-ray spectroscopy (EDS) analysis of the ash surface chemical composition.

**Figure 2:**
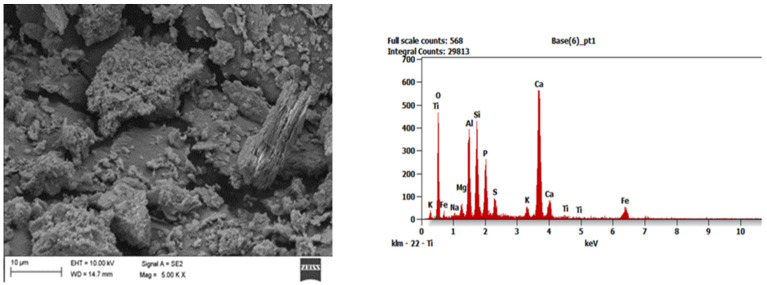
SEM image of the post-digestion sludge-based ash (combustion with kaolinite additive) and energy dispersive X-ray spectroscopy (EDS) analysis of the ash surface chemical composition.

**Figure 3:**
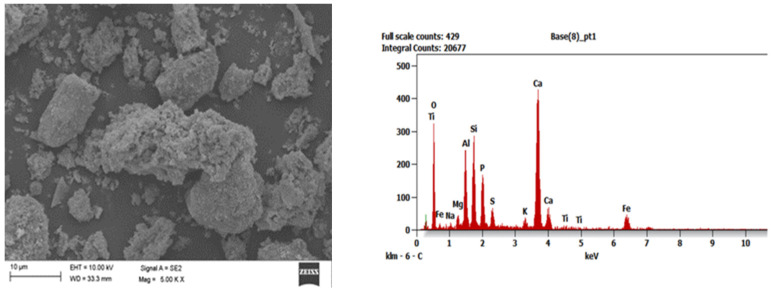
SEM image of the post-digestion sludge-based ash (combustion with halloysite additive) and energy dispersive X-ray spectroscopy (EDS) analysis of the ash surface chemical composition.

To identify the morphological structure of the obtained adsorption materials, tests were carried out with the use of a scanning electron microscope (SEM) Zeiss Supra 35 (Carl Zeiss AG, Aalen, Germany), which was equipped with the EDAX Energy dispersive X-ray spectroscopy (EDS) system (EDAX, Mahwah, NJ, USA) and enabled to analyze the chemical composition in micro-areas. This examination was performed for all series of samples after their combustion. The tests were carried out in the Faculty of Mechanical Engineering of the Silesian University of Technology (Gliwice, Poland). In the SEM images one can generally observe that the addition of aluminosilicates limiting ash agglomeration during the combustion process makes the ash microstructure more heterogeneous (exemplary Figure 1, Figure 2 and Figure 3). 

## 4. Discussion

The results of experimental research aimed at identifying the adsorption capacity of selected waste products, namely, post-digestion sludge ashes A1, A2, and A3, in relation to selected nutrients of potential importance in agriculture are presented.

Among the potential factors affecting the produced adsorbent structures, one should take into account, first of all, the composition of the original wastewater, the combinations of process parameters in the biogas production technology used in the installation integrated with the wastewater treatment plant system (analyzed in detail in authors’ other work [5]). Other important factors are the structure of the obtained ash, effects of aggregation, agglomeration, 3-D structure enabling effective contact of the leaching/extracting solution, spatial penetration for a limited interphase contact time to achieve practical extraction, and adsorption equilibrium conditions. Other phenomena like plugging certain open spaces available for the solution, attrition, and aggregation also contribute to the product properties. Self-establishing balances between adsorption and desorption (solvent extraction), composition of multi-component solutions used and other parameters, directly or indirectly influencing the final effects of the process should also be considered.

The aim of the authors was to present the general possibility of using this type of waste substance as an effective adsorptive material matrix in relation to selected chemical compounds. Moreover, it was generally shown, that depending on the combustion process conditions, in particular the co-presence of accompanying substances, i.e., additives in the form of kaolinite and halloysite, material structures are produced under the conditions of thermochemical reactions with experimentally identified, clearly better adsorption abilities in relation to some adsorbates.

It should also be emphasized at this point, that these are approximate results only, presented primarily to demonstrate the possibility of using this type of adsorbents, for example, in agriculture, intentionally as the components of soil substitutes with the adsorbed nutrients (slow release into the soil structure, possibility of cyclic re-adsorption and re-desorption, thus soil stabilization against some accidental nutrients overdosing and against some instant releasing peaks occurrence).

The tests were carried out with the use of an aqueous multicomponent Ion mixtures, which may affect the ionic strength of the S1–S5 solutions, ion activity coefficients in these systems and other physicochemical factors directly or indirectly affecting the conditions of the adsorption, desorption (extraction) phenomena, and their final practical effects.

The specific structure of the adsorbent undoubtedly also plays a key role, related both to its spatial structure (unstable aggregates, agglomerates) and to its origin, which determine the chemical composition and spatial distribution of individual components, functional groups at the surface, etc. If they are present on the outer surfaces of the grains, the mass transfer in the solvent extraction process from the solid may take place mainly as a result of the convection-diffusion process that is dominant in these conditions. On the other hand, in the case of natural distribution of a part of the substance inside the capillary-porous structures (practically no effective convection possibility), from the theoretical point of view the only mass transfer mechanism may be relatively slow diffusion in stationary liquid volumes enclosed in the 3D adsorbent structures [47]. This can explain the different extraction time of the initial substance content from the adsorptive structures, affecting the modification of the adsorption equilibrium in the system as a result of presence of an oppositely directed stream of desorbed (extracted) substances into the liquid system (negative *q*_e_ values observed in Table 4, Table 5, Table 6, Table 7, Table 8 and Table 9).

It is a specific feature of this type of structure that, immediately after forming during combustion, in its composition certain chemical components are present that can be transported to the solution or soil directly contacting the “fresh adsorbent” and based on solvent extraction mechanisms.

It is also necessary to consider the possibility of reaching only a practical (limited by technological process reasons) equilibrium during preliminary washing (25 times replacement of fresh distilled water in the batch-mode cross-extraction system during the preparation of the adsorbents A1–A3 before the actual adsorption tests). This practical equilibrium can be different than the theoretical physicochemical adsorption–desorption equilibrium. This could result in a slightly delayed in time, but still possible removal of the investigated components, naturally present in the considered bulk adsorption material. These components were responsible for such a high value of the mass flux to the solution, thus the resultant adsorption effect became opposite (desorption/extraction, according to the convention used, interpreted as negative adsorption *q*_e_), which in many cases was noticeable in Table 4, Table 5, Table 6, Table 7, Table 8 and Table 9. It can also be assumed, that the combined dissolution and diffusion processes, forming a diffusion boundary layer in the adsorbent structure, influenced the slow release of these components from the deeper layers of the adsorption material. This effect of the phenomena determining the reduction of the intensity of diffusion mass transport phenomena during desorption (solvent extraction) can, however, be used when designing the composition and structure of an adsorbent intended, for example, for a slow release of nutrients into the soil in practical, agricultural applications. Under such conditions this lag-time can, however, be regarded advantageous.

The pore size distribution directly determining the inert matrix of the adsorption material is an equally important parameter characterizing a given adsorbent structure. In this case, moreover, it is important whether the pores exhibit a passage structure or not, as they are a closed system at the end. It should be emphasized that in dynamic conditions associated with intensive shaking of the two-phase solid–liquid system, which were used during laboratory preliminary tests of solvent extraction and then adsorption, periodic and stochastic in nature closing (clogging) and opening of the transport capillary system elements may be important. It is mainly due to the periodic change in configuration and stochastic spatial structures of aggregates as a result of the dynamic interaction of the collision processes between dispersed adsorbent particles. This, depending on the intensity of shaking, also determines the effects of abrasion and aggregation in the population of sorbent particles. In dispersed systems theory (population balances) these are examples of population effects in a general population balance model affecting the resulting particle size distribution of the adsorbent.

In such an environment, a dynamic equilibrium is usually established between abrasion, attrition, breakage and, on the other hand, the phenomena of aggregation, agglomeration, which influences stochastic structural changes/rearrangements of aggregates in the dynamic (shaking) environment of solvent extraction from the solid, as well as in the dynamic environment (shaking) during adsorption. It is, however, rendering of the potential production processes (adsorption onto the structures of the obtained ashes, which will then be added to, e.g., a soil substitute for direct application in agriculture). Desorption of nutrients under static conditions (no shaking and natural soil moisture environment) should be, however, the subject of additional studies identifying the influence of the parameters of the modified final application environment on the desorbed mass stream of the investigated (previously adsorbed) nutrients important for the proper growth of crops under natural biosphere conditions. Model identification of this type of dependency is, in turn, a condition for full optimization work. This should thus concern, according to Circular Economy standards and guidelines, both the technical issues of the synthesis of the best adsorption structures (combustion with or without additives) in relation to selected adsorbates, as well as the conditions for the subsequent, effective, selective adsorption of the desired components on the inert matrix of the sorption material with their consecutive controlled desorption in biosphere.

It should be, however, taken into account that the raw sewage sludge represents a relatively complex structure. Such structure reflects both the original composition and types of the substances discharged into the environment, the natural and spontaneous decomposition processes taking place in the meantime, targeted biotechnological processes in the plant itself, and the complex influence of various combinations of technological process parameters in wastewater treatment plant (e.g., [5]). During the combustion process, high-temperature reactions take place that modify the surface functional groups, and thus alter the chemical structure of the surface in general. The porosity and the specific surface of the resulting ash material are also altered. In particular, during combustion at high temperatures of post-digestion sewage sludge with the nanostructural additives halloysite and kaolinite, there is a possibility of chemical transformation of these components and possible interactions with the complex residue biological material of the raw post-digestion sewage sludge [35,48,49,50].

Also, on the basis of the test results reported in the literature [35,48,49,50] it can be seen, that the increased fraction of sewage sludge in the combustion material causes an increased diversity, as well as the concentration of various surface functional groups. This, together with the available surface area of interfacial contact, is probably mainly responsible for the enhanced adsorption effects. The literature mentions an example of the formation of various mineral structures under the conditions of the pyrolysis process [35,48,49,50]. Especially specific chemical composition of the incinerated material affects the resulting Ash Fusion Temperatures representing some ash properties, directly or indirectly affecting the adsorption abilities [51].

For example, in the case of phosphate(V) ions, the presence of surface functional groups in the adsorbent structures, as well as the presence of metal elements is a key factor for their adsorption. The surface charge is also an important parameter for the adsorption process effect. It is also noted in the literature, that the presence of Ca, Al, Fe, Mg in the adsorbent structures is beneficial for the adsorption of phosphate(V) ions. Their oxides can potentially interact with phosphate(V) ions, e.g., in complexing reactions, where the complex compounds formed are then bound to the surface on the tested biochar. In the studies described in the literature, a partial extraction of Ca, Al, Fe, Mg from the biochar to the surrounding solution was also noted, which enabled the reaction with PO_4_^3−^ and the synthesis of insoluble phosphates(V), such as Ca_3_(PO_4_)_2_, CaHPO_4_, AlPO_4_, FePO_4_, MgHPO_4_ and Mg(H_2_PO_4_)_2_ [35,48,49,50].

The literature presents the results of studies confirming the significant influence of the presence of Si, Ca, Al, and Fe in the surface structures of the adsorbent, which fulfill the complex function of active sites in the selective adsorption of phosphates(V) (about 5-fold increase in PO_4_^3−^ adsorption, from 0.65 mg/g after removal compounds with these elements up to 2.99 mg/g in their presence) [35,48,49,50].

On the basis of these, one can thus explain the observed beneficial effect of the additives of halloysite and kaolinite for post-digestion sludge combustion with regard to the adsorption properties of the obtained ash in relation to phosphate(V) ions. In the case of the analyzed adsorbents, the undertaken initial extraction (leaching) should be emphasized, which, apart from the deliberate removal of NO_3_^−^, K^+^, Na^+^, PO_4_^3−^, SO_4_^2−^, Cl^-^ ions, was on the other hand responsible for the simultaneous removal of Si, Ca, Al, and Fe compounds being, however, important and advantageous for effective adsorption of phosphates.

The higher PO_4_^3−^ adsorption capacity of samples resulting from post-digestion sewage sludge combustion together with halloysite or kaolinite can therefore be explained by the formation of significantly higher number of complex mineral structures (compared with combustion of “pure” sludge) in a high-temperature combustion process, and thus more effective, chemical bounds-based retention of Si, Al in the adsorbent structure (these are, for example, components of chemically pure halloysite) and Fe (possible contamination of halloysite) in the complex mineral structures. These conclusions can be also confirmed by SEM and EDS observations, indicating higher concentration of Si, Al, and Fe in post-digestion sludges combusted with halloysite and kaolinite additives (Figure 1, Figure 2 and Figure 3). Due to limited solubility, the inability to extract them from the structures of the adsorbents thus formed turns out to be advantageous from the point of view of producing specific surface (adsorptive) properties of the ash samples towards phosphates. 

The complex chemical composition of the adsorbates solutions used in the research should also be taken into account in the analysis. First of all, it is the simultaneous presence of various ions in solutions, when they all affect the resultant ionic strength of the solution. Moreover, there are various interactions between different ions in the solution, increasing or decreasing the adsorbent efficiency of each of the adsorbate individually (various combinations adsorbent–multicomponent solutions tested).

The own results of the adsorption of phosphate(V) ions (0.0136–0.2411 mg/g–ash without additives, 0.3206–1.0879 mg/g for post-digestion sludge combustion with kaolinite and 0.4392–1.1659 mg/g for sludge combustion with halloysite) obtained in the presented tests are in general magnitude comparable to the results related to unmodified sludge-based adsorption materials (e.g., 2.78 mg/g total phosphorus for unmodified sewage sludge carbon, for carbonized sludge 3.8 mg/g, but after modification with pyrolusite it increased up to 10.78 mg/g, with the addition of iron–even up to 111 mg/g, the addition of zirconium increases it up to 27.55 mg P/g). Other studies presented in the literature showed a broad possibility of changing the maximum adsorption capacity in the range of 4.2 mg P/g–90.1 mg P/g [35,48,49,50].

The research results presented in the literature emphasized the decisive influence of the source of the wastewater, the applied technological treatment processes, and the subsequent modification (postprocessing–functionalization) of the sludge. The maximum adsorption capacity for phosphate(V) ions was as high as 303.49 mg/g. It was explained by the concentration of metal oxides and surface functional groups directly bound as an unintended effect of technological processes of wastewater purification. Such an accumulation of surface adsorption centers indirectly influences the dominance of chemical adsorption mechanisms in this physicochemical system, as presented in the literature [35,48,49,50]. However, this may be, on the other hand, some form of restriction in application of such materials in agriculture (high metals concentration).

The predetermined time of interphase contact during adsorption is also important. In the presented research it was tested from the point of view of practical equilibrium with the possibility of a limited time of interphase contact in industrial conditions.

It should be emphasized, that the lower adsorption capacity observed in the presented tests may result both from the study of multicomponent solutions (as may be the case in the actual process of introducing multicomponent mineral fertilizers into the soil, and thus the need to verify the operation of the adsorption system in such an imposed sorption environment) and the promoted idea of Direct Circular Economy direct use of the obtained products, without deliberately supplementing them with ingredients promoting sorption processes, which at the same time increases their market price and this way possibly reduces the range of possible use in agriculture.

In general, due to the limited number of obtained measurement data, their dispersion and the resulting sometimes low *R*^2^ values indicating directly the poor quality of the model, the authors would like to emphasize that the modelling results should be treated only as preliminary and estimates. Thus, a more detailed theoretical understanding of the problem, including examination of a larger number of laboratory data (which is planned by the authors for the near future) and in-depth statistical verification of their compliance with the predictions of various theoretical models should provide a much wider range of information on potential sorption mechanisms involved and possibly their interdependencies or changes in different process conditions.

## 5. Conclusions

The most spectacular and clearly favorable results related to the influence of nanostructural additives in the process of sludge combustion and formation of sorption surfaces under high temperature conditions were obtained in the case of sorption-based separation of phosphate(V) ions (an increase from 1.13% to 61.24% with the addition of kaolinite and up to 76.19% with addition of halloysite).

The introduction of nanostructural additives to the combustion process resulted in complex interactions in heterogeneous processes and reaction schemes, potentially related to the modification of the surface morphology and the surface functional groups. The clear effects of increasing the sorption capacity of phosphate(V) ions (as well as others) are directly related to the presence of kaolinite or halloysite in the environment of high-temperature heterogeneous reactions in the analyzed systems. It can be hypothesized that nanostructured systems introduced into the reacting environment significantly increased specific surface area of interfacial contact and a better spatial distribution of "contaminants" (Si, Al, Fe), which, potentially immobilized as a result of chemical reactions and thus more difficult to remove in the preliminary physical extraction stage, turned out to be of key importance in the processes of phosphate(V) sorption, as was independently confirmed in SEM / EDS analyses.

The innovative technological Circular Economy approach for the nutrients adsorption by the post-digestion sewage sludge-based ash co-formed with some nanostructural additives, is a fragment of the authors’ general concept. It is aimed at optimizing the multi-stage conversion of residues from wastewater treatment. The “leading concept” was to reduce the environmental nuisance of originally, potentially emitted greenhouse gases to relatively less harmful ones (CO_2_ instead of methane) through a rational chain of biotechnological and thermal transformations. Further work of the authors will be focused on extending the areas of application of the functionalized ashes and adsorbents, derived from other types of wastewater treatment processes and plants, for example from different industrial wastewaters. Depending on the specific composition of such wastewater and different soil cleanliness requirements, these can be used in degraded or agricultural areas. The proposed technological approach to the method of producing the thermally functionalized “ashes–reusable adsorbents” can be used practically for all possible types of sewage treatment plants and biogas plants, industrial, agricultural, municipal, and mixed ones. Even in potentially extreme contaminated cases, thermal conversion products can be effectively employed for the controlled fertilization of heavily degraded post-industrial land areas as an element increasing, through targeted fertilization (N, P, K) the growth intensity of local plant biomass, and thus directly increasing the overall effectiveness of the phytoremediation processes. On the basis of the obtained original data, it can be concluded, that the combustion and co-combustion of the discussed waste post-digestion sludge should not be connected with the common technological/exploitation problems in the combustion chamber (the so-called “ash-related issues”). This is a direct result of the high AFT values and the moderate chlorine concentration in these sludges.

In the vast majority of the analyzed measurement cases, the theoretical analysis indicates a better statistical compliance of the Freundlich isotherm model. Its theoretical interpretation indicates the presence of an imperfect adsorption process on the material with adsorption properties produced under high temperature conditions. The heterogeneous surface morphology predicted by the Freundlich isotherm model is also visible in the SEM images (Figure 1, Figure 2 and Figure 3). Based on the Freundlich model, it is also possible to suggest the phenomena of multilayer adsorption as the dominant sorption mechanism characteristic for the obtained adsorbents based on ashes from the combustion of sludge after anaerobic fermentation (with or without the inorganic nanostructural additives studied in this work).

The obtained measurement data indicate that the addition of kaolinite or halloysite caused the formation of adsorptive structures increasing the separation capacity. Especially in the case of phosphate(V) ions this experimentally confirmed affinity was high. This may justify the potential use of this type of innovative environmentally inert adsorbent for the slow release (after adsorption) of this component into the soil in a controlled manner. It should also be emphasized here, that the addition of kaolinite or halloysite has a positive effect on the entire process of combustion of the post-digestion sludge.

The observed negative adsorption values are mainly caused by the delayed desorption (extraction, leaching) process of compounds naturally present in the ash structure (net effect of simultaneous desorption and adsorption phenomena). It is mainly related to the phenomena of convective-diffusional or only diffusional mass transport in the complex spatial structure of ash agglomerates.

The obtained original results can be used in the further studies and detailed design works related to the suggested innovative pro-ecological management of the post-digestion sludge-derived ashes according to Circular Economy standards as adsorbents of fertilizing compounds in agricultural applications (for their slow release from environmentally inert carriers). It should be emphasized, once again, that the introduced nanostructured additives, which are the key and original aspect of this innovative technological approach, have a beneficial effect on many stages of this process, both the course of the co-combustion process itself and the reactions environment leading to the formation of an adsorptive surface enabling further use of such ash type in agriculture.

## Figures and Tables

**Table 1:** Chemical composition of dewatered post-digestion sludge (data for 2021).

Parameter	Min	Max	Mean
pH	7.4	12.5	10.7
Dry mass (d.m.) [%]	15.3	22.2	19.2
Organic substances [% d.m.]	20.3	63.8	45.6
Total N [% d.m.]	1.58	5.16	3.33
NH_4_ [% d.m.]	0.10	0.84	0.60
Total P [% d.m.]	2.06	2.95	2.39
Ca [% d.m.]	2.75	17.8	9.48
Mg [% d.m.]	0.50	1.64	0.76
Cd [mg/kg d.m.]	1.27	1.68	1.54
Cu [mg/kg d.m.]	132	270	220.5
Ni [mg/kg d.m.]	16.7	28	20.62
Pb [mg/kg d.m.]	25.1	39.2	30.85
Zn [mg/kg d.m.]	364	1278	660
Hg [mg/kg d.m.]	0.19	0.93	0.44
Cr [mg/kg d.m.]	24.3	59.9	36.5

**Table 2:** Proximate and ultimate analysis of post-digestion sewage sludge.

Parameter	Basis	Unit	Post-Digestion Sewage Sludge
Moisture	a.r.	wt%	1.8
Ash	d.b.	wt%	58.3
HHV	d.b.	MJ/kg	13.69
LHV	a.r.	MJ/kg	12.54
Cl	d.b.	wt%	0.089
C	d.b.	wt%	31.77
H	d.b.	wt%	4.05
N	d.b.	wt%	3.71
S	d.b.	wt%	1.34

a.r.–as received; d.b.–dry-basis.

**Table 3:** Chemical composition of the post-digestion sewage sludge ashes (wt.%) and corresponding AFTs (Ash Fusion Temperatures).

Chemical Component	Ash from Post-Digestion Sewage Sludge A1	Ash from Post-Digestion Sewage Sludge with Halloysite A3	Ash from Post-Digestion Sewage Sludge with Kaolinite A2
Cl	0.20	0.18	0.19
SO_3_	4.20	4.48	4.24
K_2_O	1.87	2.05	1.83
SiO_2_	30.20	31.20	31.80
Fe_2_O_3_	5.80	5.94	5.44
Al_2_O_3_	7.36	7.47	7.80
Mn_3_O_4_	0.09	0.10	0.09
TiO_2_	0.58	0.61	0.55
CaO	29.49	28.25	28.66
MgO	2.83	2.73	2.78
P_2_O_5_	14.72	14.30	13.99
Na_2_O	2.07	2.10	1.97
BaO	0.03	0.03	0.04
SrO	0.06	0.05	0.05
Ash fusion temperature in reducing/oxidizing atmosphere, °C			
Initial deformation temperature (IDT)	1240/1200	1220/1160	1230/1190
Softening temperature (ST)	1290/1260	1260/1250	1250/1240
Hemisphere temperature (HT)	1350/1310	1340/1300	1340/1300
Flow temperature (FT)	1420/1400	1390/1360	1380/1340

**Table 4:** Experimental data concerning adsorption of NO_3_^−^ ions from aqueous solutions S1–S5 on three types of waste post-digestion sludge ash-derived adsorbents A1–A3.

No	Ash without Any Additives (Reference Ash) A1					Ash Derived from Post-Digestion Sludge Combustion with Kaolinite A2					Ash Derived from Post-Digestion Sludge Combustion with Halloysite A3				
Concentration test	S1	S2	S3	S4	S5	S1	S2	S3	S4	S5	S1	S2	S3	S4	S5
1 [mg/dm^3^]	58.1	120.4	172.8	243.2	296	58.1	120.4	172.8	243.2	296	58.1	120.4	172.8	243.2	296
2 [mg/dm^3^]	58	120.4	173	243.6	295.6	58	120.4	173	243.6	295.6	58	120.4	173	243.6	295.6
3 [mg/dm^3^]	58.1	120.8	173.2	244.4	295.6	58.1	120.8	173.2	244.4	295.6	58.1	120.8	173.2	244.4	295.6
**Mean C_0_** [mg/dm^3^]	**58.07**	**120.53**	**173**	**243.73**	**295.73**	**58.07**	**120.53**	**173**	**243.73**	**295.73**	**58.07**	**120.53**	**173**	**243.73**	**295.73**
4 [mg/dm^3^]	50.8	107	179.6	211.8	312.4	52.1	114	166.4	261.6	304.4	54.2	111.8	166	252.8	318.4
5 [mg/dm^3^]	51	107.4	179.6	211.8	312.8	52.5	114	166.8	262.4	304.4	54	112	166	251.2	319.6
6 [mg/dm^3^]	52.1	107.6	179.6	211.4	313.6	52.5	114	166.8	262.8	304.8	53.7	112	166	252.4	319.6
**Mean C_e_** [mg/dm^3^]	**51.30**	**107.33**	**179.6**	**211.67**	**312.93**	**52.37**	**114**	**166.67**	**262.27**	**304.53**	**53.97**	**111.93**	**166**	**252.13**	**319.2**
*q_e_* * [mg/g]	0.0395	0.0770	−0.0385	0.1871	−0.1003	0.0332	0.0381	0.0369	−0.1081	−0.0513	0.0239	0.0502	0.0408	−0.049	−0.1369
Removal (%)	11.65	10.95	–	13.16	–	9.82	5.42	3.66	–	–	7.06	7.13	4.05	–	–
Relative removal **	1	1	–	1	–	0.843	0.495	–	–	–	0.606	0.651	–	–	–

* Negative values indicating a higher desorption (extraction) effect of the selected component into the solution than its adsorption from the solution; ** The relative values (for each solution S1–S5 individually) based on ash A1 as a reference level.

**Table 5:** Experimental data concerning adsorption of K^+^ ions from aqueous solutions S1–S5 on three types of waste post-digestion sludge ash-derived adsorbents A1–A3.

No	Ash without Any Additives (Reference Ash) A1					Ash Derived from Post-Digestion Sludge Combustion with Kaolinite A2					Ash Derived from Post-Digestion Sludge Combustion with Halloysite A3				
Concentration test	S1	S2	S3	S4	S5	S1	S2	S3	S4	S5	S1	S2	S3	S4	S5
1 [mg/dm^3^]	51	65	84	108	122	51	65	84	108	122	51	65	84	108	122
2 [mg/dm^3^]	51	65	85	109	123	51	65	85	109	123	51	65	85	109	123
3 [mg/dm^3^]	51	65	85	109	123	51	65	85	109	123	51	65	85	109	123
**Mean C_0_** [mg/dm^3^]	**51**	**65**	**84.67**	**108.67**	**122.67**	**51**	**65**	**84.67**	**108.67**	**122.67**	**51**	**65**	**84.67**	**108.67**	**122.67**
4 [mg/dm^3^]	44	49	74	107	121	41	52	74	98	122	42	54	70	94	121
5 [mg/dm^3^]	45	50	75	107	122	41	52	75	99	122	42	54	71	94	121
6 [mg/dm^3^]	45	51	75	107	121	42	52	75	99	123	42	55	71	95	122
**Mean C_e_** [mg/dm^3^]	**44.67**	**50**	**74.67**	**107**	**121.33**	**41.33**	**52**	**74.67**	**98.67**	**122.33**	**42**	**54.33**	**70.67**	**94.33**	**121.33**
*q_e_* * [mg/g]	0.0369	0.0875	0.0583	0.0097	0.0078	0.0564	0.0758	0.0583	0.0583	0.0019	0.0525	0.0622	0.0817	0.0836	0.0078
Removal (%)	12.42	23.08	11.81	1.53	1.09	18.95	20	11.81	9.20	0.27	17.65	16.41	16.54	13.19	1.09
Relative removal **	1	1	1	1	1	1.526	0.867	1	6.013	0.248	1.421	0.711	1.400	8.621	1

* Negative values indicating a higher desorption (extraction) effect of the selected component into the solution than its adsorption from the solution; ** The relative values (for each solution S1–S5 individually) based on ash A1 used as a reference level.

**Table 6:** Experimental data concerning adsorption of Na^+^ ions from aqueous solutions S1–S5 on three types of waste post-digestion sludge ash-derived adsorbents A1–A3.

No	Ash without Any Additives (Reference Ash) A1					Ash derived from Post-Digestion Sludge Combustion with Kaolinite A2					Ash Derived from Post-Digestion Sludge Combustion with Halloysite A3				
Concentration test	S1	S2	S3	S4	S5	S1	S2	S3	S4	S5	S1	S2	S3	S4	S5
1 [mg/dm^3^]	30	70	107	144	174	30	70	107	144	174	30	70	107	144	174
2 [mg/dm^3^]	30	69	109	146	174	30	69	109	146	174	30	69	109	146	174
3 [mg/dm^3^]	30	69	109	144	175	30	69	109	144	175	30	69	109	144	175
**Mean C_0_** [mg/dm^3^]	**30**	**69.33**	**108.33**	**144.67**	**174.33**	**30**	**69.33**	**108.33**	**144.67**	**174.33**	**30**	**69.33**	**108.33**	**144.67**	**174.33**
4 [mg/dm^3^]	35	77	114	149	184	34	71	113	140	169	33	71	114	133	181
5 [mg/dm^3^]	35	78	115	149	184	34	71	113	140	169	33	70	114	134	180
6 [mg/dm^3^]	35	77	115	149	184	32	74	112	140	170	33	72	114	134	182
**Mean C_e_** [mg/dm^3^]	**35**	**77.33**	**114.67**	**149**	**184**	**33.33**	**72**	**112.67**	**140**	**169.33**	**33**	**71**	**114**	**133.67**	**181**
*q_e_* * [mg/g]	−0.0292	−0.0467	−0.0369	−0.0253	−0.0564	−0.0194	−0.0156	−0.0253	0.0272	0.0292	−0.0175	−0.0097	−0.0331	0.0642	−0.0389
Removal (%)	−	−	−	−	−	−	−	−	3.23	2.87	−	−	−	7.60	−
Relative removal **	−	−	−	−	−	−	−	−	1	1	−	−	−	2.353	−

* Negative values indicating a higher desorption (extraction) effect of the selected component into the solution than its adsorption from the solution; ** The relative values (for each solution S1−S5 individually) based on ash A2 used as a reference level.

**Table 7:** Experimental data concerning adsorption of PO_4_^3−^ ions from aqueous solutions S1–S5 on three types of waste post-digestion sludge ash-derived adsorbents A1–A3.

No	Ash without Any Additives (Reference Ash) A1					Ash Derived from Post-Digestion Sludge Combustion with Kaolinite A2					Ash Derived from Post-Digestion Sludge Combustion with Halloysite A3				
Concentration test	S1	S2	S3	S4	S5	S1	S2	S3	S4	S5	S1	S2	S3	S4	S5
1 [mg/dm^3^]	87.2	113	166	206	285	87.2	113	166	206	285	87.2	113	166	206	285
2 [mg/dm^3^]	87.3	113	166	207	285	87.3	113	166	207	285	87.3	113	166	207	285
3 [mg/dm^3^]	87.3	113	167	207	285	87.3	113	167	207	285	87.3	113	167	207	285
**Mean C_0_** [mg/dm^3^]	**87.27**	**113**	**166.33**	**206.67**	**285**	**87.27**	**113**	**166.33**	**206.67**	**285**	**87.27**	**113**	**166.33**	**206.67**	**285**
4 [mg/dm^3^]	93.1	133	231	204	244	124	57.7	29.7	80.1	98.6	165	178	91.1	49	85.1
5 [mg/dm^3^]	93.4	133	231	205	243	124	58.5	30.2	79.9	98.3	165	178	91	49.3	84.9
6 [mg/dm^3^]	92.9	133	231	204	244	124	57.9	30	80.3	98.6	165	178	91	49.3	85.4
**Mean C_e_** [mg/dm^3^]	**93.13**	**133**	**231**	**204.33**	**243.67**	**124**	**58.03**	**29.97**	**80.10**	**98.50**	**165**	**178**	**91.03**	**49.20**	**85.13**
*q_e_* * [mg/g]	−0.0342	−0.1167	−0.3772	0.0136	0.2411	−0.2143	0.3206	0.7955	0.7383	1.0879	−0.4534	−0.3792	0.4392	0.9186	1.1659
Removal (%)	–	–	–	1.13	14.50	–	48.64	81.98	61.24	65.44	–	–	45.27	76.19	70.13
Relative removal **	–	–	–	1	1	–	–	–	54.195	4.513	–	–	–	67.425	4.837

* Negative values indicating a higher desorption (extraction) effect of the selected component into the solution than its adsorption from the solution; ** The relative values (for each solution S1–S5 individually) based on ash A1 used as a reference level.

**Table 8:** Experimental data concerning adsorption of SO_4_^2−^ ions from aqueous solutions S1–S5 on three types of waste post-digestion sludge ash-derived adsorbents A1–A3.

No	Ash without Any Additives (Reference Ash) A1					Ash Derived from Post-Digestion Sludge Combustion with Kaolinite A2					Ash Derived from Post-Digestion Sludge Combustion with Halloysite A3				
Concentration test	S1	S2	S3	S4	S5	S1	S2	S3	S4	S5	S1	S2	S3	S4	S5
1 [mg/dm^3^]	472.5	1022.5	1302.5	1905	2097.5	472.50	1022.5	1302.50	1905	2097.5	472.50	1022.5	1302.5	1905	2097.5
2 [mg/dm^3^]	512.5	1012.5	1295	1900	2100	512.50	1012.5	1295	1900	2100	512.5	1012.5	1295	1900	2100
3 [mg/dm^3^]	507.5	1017.5	1295	1907.5	2097.5	507.50	1017.5	1295	1907.5	2097.5	507.50	1017.5	1295	1907.5	2097.5
**Mean C_0_** [mg/dm^3^]	**497.50**	**1017.50**	**1297.50**	**1904.17**	**2098.33**	**497.50**	**1017.50**	**1297.50**	**1904.17**	**2098.33**	**497.50**	**1017.50**	**1297.50**	**1904.17**	**2098.33**
4 [mg/dm^3^]	725	945	1270	1547.5	1790	693	1000	1145	1572.5	1735	613	958	1122.5	1525	1730
5 [mg/dm^3^]	732	938	1260	1547.5	1802.50	692	1000	1150	1565	1715	611	957	1112.5	1505	1725
6 [mg/dm^3^]	721	937	1270	1532.5	1785	692	1000	1145	1557.5	1697.5	610	959	1105	1510	1725
**Mean C_e_** [mg/dm^3^]	**726**	**940**	**1266.67**	**1542.50**	**1792.50**	**692.33**	**1000**	**1146.67**	**1565**	**1715.83**	**611.33**	**958**	**1113.33**	**1513.33**	**1726.67**
*q_e_* * [mg/g]	−1.3329	0.4521	0.1799	2.1097	1.7840	−1.1365	0.1021	0.8799	1.9785	2.2312	−0.6640	0.3471	1.0743	2.2799	2.1681
Removal (%)	–	7.62	2.38	18.99	14.58	–	1.72	11.62	17.81	18.23	–	5.85	14.19	20.53	17.71
Relative removal **	–	1	1	1	1	–	0.226	4.882	0.938	1.250	–	0.768	5.962	1.081	1.215

* Negative values indicating a higher desorption (extraction) effect of the selected component into the solution than its adsorption from the solution; ** The relative values (for each solution S1–S5 individually) based on ash A1 used as a reference level.

**Table 9:** Experimental data concerning adsorption of Cl^−^ ions from aqueous solutions S1–S5 on three types of waste post-digestion sludge ash-derived adsorbents A1–A3.

No	Ash without Any Additives (Reference Ash) A1					Ash Derived from Post-Digestion Sludge Combustion with Kaolinite A2					Ash Derived from Post-Digestion Sludge Combustion with Halloysite A3				
Concentration test	S1	S2	S3	S4	S5	S1	S2	S3	S4	S5	S1	S2	S3	S4	S5
1 [mg/dm^3^]	81	121	169	215	261	81	121	169	215	261	81	121	169	215	261
2 [mg/dm^3^]	82	121	171	217	264	82	121	171	217	264	82	121	171	217	264
3 [mg/dm^3^]	82	122	171	219	268	82	122	171	219	268	82	122	171	219	268
**Mean C_0_** [mg/dm^3^]	**81.67**	**121.33**	**170.33**	**217**	**264.33**	**81.67**	**121.33**	**170.33**	**217**	**264.33**	**81.67**	**121.33**	**170.33**	**217**	**264.33**
4 [mg/dm^3^]	57	122	172	212	270	66	119	173	223	267	55	109	179	222	>250
5 [mg/dm^3^]	57	123	174	213	273	66	120	174	224	269	56	110	180	224	>250
6 [mg/dm^3^]	57	123	175	214	273	67	121	174	226	270	56	111	181	225	>250
**Mean C_e_** [mg/dm^3^]	**57**	**122.67**	**173.67**	**213**	**272**	**66.33**	**120**	**173.67**	**224.33**	**268.67**	**55.67**	**110**	**180**	**223.67**	**>250**
*q_e_* * [mg/g]	0.1439	−0.0078	−0.0194	0.0233	−0.0447	0.0894	0.0078	−0.0194	−0.0428	−0.0253	0.1517	0.0661	−0.0564	−0.0389	–
Removal (%)	30.20	–	–	1.84	–	18.78	1.10	–	–	–	31.84	9.34	–	–	–
Relative removal **	1	–	–	1	–	0.622	–	–	–	–	1.054	–	–	–	–

* Negative values indicating a higher desorption (extraction) effect of the selected component into the solution than its adsorption from the solution; ** The relative values (for each solution S1–S5 individually) based on ash A1 used as a reference level.

## Data Availability

Not applicable.
